# A randomized, double-blind, placebo-controlled phase III trial of duloxetine in Japanese fibromyalgia patients

**DOI:** 10.1186/s13075-015-0718-y

**Published:** 2015-08-22

**Authors:** Masato Murakami, Kenichi Osada, Hiromichi Mizuno, Toshimitsu Ochiai, Levent Alev, Kusuki Nishioka

**Affiliations:** Department of Psychosomatic Medicine, Nihon University School of Medicine, 30-1 Oyaguchi Kamicho, Itabashi-ku, Tokyo 173-8610 Japan; Department of Neuropsychiatry, St. Marianna University School of Medicine, 2-16-1 Sugao, Miyamae-ku, Kawasaki, 216-8511 Japan; Shionogi & Co. Ltd., 12F, Hankyu Terminal Bldg, 1-4 Shibata 1-chome, Kita-ku, Osaka 530-0012 Japan; Eli Lilly Japan K.K., Sannomiya Plaza Building, 7-1-5 Isogamidori, Chuo-ku, Kobe, 651-0086 Japan; Institute of Medical Science, Tokyo Medical University, 6-1-1 Shinjuku, Shinjuku-ku, Tokyo 160-8402 Japan

## Abstract

**Introduction:**

Fibromyalgia is characterized by widespread pain and is often accompanied by accessory symptoms. There are limited treatment options for this condition in Japan. Therefore, we conducted a phase III study to assess the efficacy and safety of duloxetine in Japanese patients with fibromyalgia.

**Methods:**

This randomized, double-blind, placebo-controlled, parallel-group trial was conducted in Japan. Outpatients who met the American College of Rheumatology 1990 criteria for fibromyalgia and whose Brief Pain Inventory (BPI) average pain score was ≥4 were randomized to duloxetine 60 mg or placebo once daily for 14 weeks. The primary efficacy measure was the change in the BPI average pain score from baseline. Secondary efficacy, quality of life (QoL), and safety outcomes were also evaluated. Mixed-effects model repeated-measures (MMRM) analysis and last observation carried forward (LOCF) analysis of covariance were used to evaluate the primary efficacy measure.

**Results:**

Overall, 393 patients were randomized to receive either duloxetine (n = 196) or placebo (n = 197). The MMRM analysis revealed no significant difference between duloxetine and placebo regarding the change in BPI average pain scores at week 14. Based on LOCF analysis, a statistically significant improvement in the change in BPI average pain scores at week 14 was observed for patients treated with duloxetine compared with placebo. Duloxetine treatment was associated with improved outcomes in nearly all secondary and post hoc analyses. The treatment was generally well tolerated. Somnolence, nausea, and constipation were the most common treatment-emergent adverse events in the duloxetine group. The discontinuation rates due to treatment-emergent adverse events were similar in both groups.

**Conclusions:**

Although the MMRM analysis did not demonstrate superiority of duloxetine over placebo, duloxetine treatment was associated with improved outcomes in secondary and post hoc analyses of the mean change in the BPI average pain score and most of the secondary outcomes, including analgesia and QoL. Duloxetine treatment was safe and well tolerated. These results suggest that duloxetine treatment could be associated with improvements in pain relief and QoL in Japanese patients with fibromyalgia.

**Trial registration:**

ClinicalTrials.gov Identifier: NCT01552057. Registered 9 March 2012.

## Introduction

Fibromyalgia is a disorder characterized by widespread pain. In addition to widespread pain, patients with fibromyalgia frequently experience other troublesome symptoms, such as fatigue, sleep disturbances, and cognitive disturbances; other specific painful conditions such as chronic headache, temporomandibular disorders, and irritable bowel syndrome; and psychiatric comorbid conditions, including anxiety and depression. Furthermore, fibromyalgia often has negative effects on personal relationships, careers, and daily activities [1–7]. According to the 1990 American College of Rheumatology (ACR) criteria for the classification of fibromyalgia [2], the diagnosis consists of two components: presence of widespread pain for at least 3 months and presence of tenderness at 11 or more of the 18 specific tender point sites. The presence of associated symptoms was included as a required component of fibromyalgia diagnosis in the preliminary diagnostic criteria for fibromyalgia published by the ACR in 2010 [8] and in revised form in 2011 [9].

In an epidemiological survey conducted in 2004, the Ministry of Health, Labour and Welfare of Japan reported a prevalence of fibromyalgia in Japan of 1.7 %, accounting for approximately 2 million individuals [1, 10], which is similar to the prevalence (2.0 %) reported in a U.S. study [3]. This prevalence rate in Japan is consistent with the results of an internet survey conducted in 2011 [6]. The male-to-female ratio of affected individuals was 1:4.8, and the mean (SD) age was 51.5 (16.9) years. Thus, in Japan, fibromyalgia predominantly affects middle-aged and elderly women. However, there are few clinicians in Japan who are aware of this disease and are able to diagnose it correctly. Reportedly, it can take approximately 4 years to establish a definitive diagnosis of fibromyalgia in Japan [1, 10]. In the same survey, patients reported dissatisfaction with current treatment.

Three agents (duloxetine, milnacipran, and pregabalin) have been approved for treatment of fibromyalgia in the United States [11]. Conversely, in Japan, the *Diagnostic Guidelines for Fibromyalgia 2013* document [1] lists a wide variety of agents for treatment, including antidepressants (tricyclic antidepressants, serotonin (5-HT) and noradrenaline (NA) reuptake inhibitors, and selective 5-HT reuptake inhibitors), anticonvulsants, and Neurotropin (an extract obtained from cutaneous tissue of rabbits inoculated with vaccinia virus; Nippon Zoki Pharmaceutical Co., Ltd., Osaka, Japan). Currently, however, only pregabalin is approved for the treatment of fibromyalgia in Japan, and the lack of other options is considered an obstacle in the treatment of this condition. Both 5-HT and NA have been thought to mediate the endogenous pain-inhibitory mechanisms via the descending pain inhibitory pathways in the brain and spinal cord [12, 13]. In chronic pain states, the net inhibitory effect of these monoamines appears to be reduced or lost, shifting the descending pain modulatory system from a state of inhibition toward a state of pain facilitation [14]. Duloxetine is a potent and selective inhibitor of 5-HT and NA reuptake in vitro and in vivo in the central nervous system (CNS). The analgesic effects of duloxetine are believed to result from increased activity of 5-HT and NA within the CNS, presumably either by enhancing the descending pain inhibitory pathways in the brain and spinal cord or via other unknown CNS actions [15, 16]. Clinical studies conducted in other countries in patients with fibromyalgia have demonstrated the safety and efficacy of duloxetine compared with placebo [17–22]. Furthermore, duloxetine has been approved for the treatment of pain associated with diabetic peripheral neuropathic pain, chronic pain caused by osteoarthritis, and chronic low back pain.

In Japan, duloxetine has been approved for treatment of major depressive disorder and diabetic peripheral neuropathic pain after its efficacy for these conditions was validated in phase III studies [23, 24]. Despite the lack of clinical study evidence of the efficacy of duloxetine among Japanese patients with fibromyalgia, there is a strong demand from the Japan College of Fibromyalgia Investigation and the Ministry of Health, Labour and Welfare to develop an indication for using duloxetine to treat fibromyalgia. Therefore, we conducted a phase III study to formally assess the efficacy and safety of duloxetine at 60 mg once daily compared with placebo in Japanese patients with fibromyalgia.

## Methods

### Overview

This randomized, multicenter, double-blind, placebo-controlled, parallel-group, phase III trial was conducted in 42 outpatient clinics and hospitals (listed in the Acknowledgments) in Japan between March 2012 and December 2013. An institutional review board for each site (listed in the Appendix) approved the protocol, and all patients provided written informed consent before study commencement. The study was conducted in compliance with the International Conference on Harmonisation Good Clinical Practice guidelines. All monitoring activities for this study were outsourced to SRL Medisearch, Inc., Tokyo, Japan. This study was registered at ClinicalTrials.gov under the identifier NCT01552057 on 9 March 2012. There were no changes to the methods or planned endpoints after study initiation.

### Eligibility criteria

The criteria used in a previous study of duloxetine [25] were adopted. Briefly, male and female outpatients aged between 20 and 75 years who met the ACR 1990 criteria for fibromyalgia [2] and had a Brief Pain Inventory (BPI) average pain score ≥4 [26, 27] at visits 1 and 2 were included. Exclusion criteria were as follows: past duloxetine treatment; serious or medically unstable disease, clinically significant abnormal laboratory values, or abnormal electrocardiogram (ECG) findings; pain caused by non-fibromyalgia diseases; poorly controlled thyroid dysfunction; rheumatoid, inflammatory, or infectious arthritis; autoimmune disorders other than thyroid dysfunction; psychiatric disorders other than major depressive disorder within the past year; and suicidal tendencies as assessed using the Columbia-Suicide Severity Rating Scale (C-SSRS) [28].

Patients were prohibited from using analgesics and drugs with analgesic effects, including nonsteroidal anti-inflammatory drugs, anticonvulsants, pregabalin, neurotropin, anesthetics, opioids, and adrenocorticosteroids. The use of analgesics for up to 3 consecutive days and for up to a total of 10 days was permitted only for the treatment of adverse events (AEs). Coadministration of acetaminophen at doses up to 1500 mg/day was permitted to treat AEs and as rescue treatment for fibromyalgia, except on the day before efficacy was evaluated after visit 2 and until just before the evaluation. The use of prophylactic aspirin at doses up to 325 mg/day to prevent cardiac events was also permitted. Patients taking mood-affecting drugs such as antidepressants, sedatives, and benzodiazepines were also excluded. Zopiclone and zolpidem were the only hypnotics permitted during the study, as long as their use began before the participant entered the screening phase (i.e., before visit 1), without dosing changes. Tender point injections and nerve blocks were to be stopped before visit 1. Non-drug therapies (e.g., exercise therapy and cognitive behavioral therapy) received at least 14 days before visit 1 were permitted during the study, as long as no changes were made.

### Study design

**Fig. 1:**
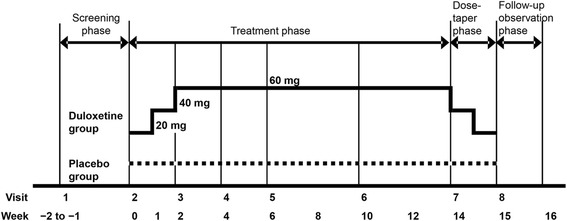
Study design

This randomized, multicenter, double-blind, placebo-controlled, parallel-group phase III trial consisted of four phases. The study lasted 17–18 weeks and included a 1- to 2-week screening phase, a 14-week treatment phase, a 1-week dose-tapering phase, and a 1-week follow-up observation phase (Fig. 1). After the screening phase, patients were assigned randomly to receive duloxetine or placebo in a 1:1 ratio, using a web-based patient registration system (ACRONET Corp., Tokyo, Japan) with a stochastic minimization procedure. The following allocation factors were used: (1) BPI average pain score at visit 2 (<6 vs. ≥6) and (2) presence or absence of concomitant major depressive disorder diagnosed on the basis of the M.I.N.I. International Neuropsychiatric Interview–Japanese version 5.0.0 [29]. It was ensured that the maximum between-group difference in the number of subjects in each medical institution did not exceed two. Blinding was maintained until the end of the study by the person responsible for the study drug assignment.

Duloxetine or placebo was orally administered once daily after breakfast on a double-blind basis. In the duloxetine group, patients received 20 mg for 1 week, followed by 40 mg for 1 week and then 60 mg for 12 weeks during the treatment phase. In the placebo group, subjects received placebo for 14 weeks throughout the treatment phase. Down-titration was performed after completion of the treatment phase or if the patient discontinued after at least 2 weeks of treatment with duloxetine (i.e., if the patient was taking 40 or 60 mg at the time of discontinuation). The drug allocation controller confirmed the study drugs were undiscernible in terms of appearance, packaging, and labeling, and mock titration of placebo pills was also performed to maintain blinding. Only the drug allocation controller was aware of the type of drugs being dispensed.

### Outcome measures

#### Primary efficacy outcomes

The primary efficacy measure was the change in the BPI average pain score from baseline (visit 2) to the end of the 14-week treatment phase (visit 7). The pain score was based on a scale from 0 (no pain) to 10 (pain as bad as patient can imagine).

#### Secondary efficacy outcomes

Secondary outcomes included the “worst pain severity,” “least pain severity,” and “pain right now” items of the BPI; pain interference with seven daily activities (general activity, walking, work, mood, enjoyment of life, relationships with others, and sleep); data reported in patient diaries; and scores on the Patient Global Impression of Improvement (PGI-I) and the Clinical Global Impressions–Global Improvement (CGI-I) [30]. Pain interference was assessed on a 0 (does not interfere) to 10 (completely interferes) rating scale. Items corresponding to the average and worst pain severity items of the BPI were recorded daily by each patient in the patient diary. The average weekly values were calculated based on the diary. PGI-I and CGI-I [30] were assessed separately by the subjects and physicians on a scale ranging from 1 (very much better/improved) to 7 (very much worse) regarding disease improvement from baseline.

#### Health outcomes

Patients responded to the Fibromyalgia Impact Questionnaire (FIQ; Japanese version) [31, 32], consisting of 20 questions about the symptoms and discomforts of fibromyalgia. Responses were summed to yield a total score ranging from 0 (no impact) to 100 (maximum impact).

Using the 36-Item Short-Form Survey (SF-36; Japanese version 2) [33, 34], patients assessed their health status by answering 36 questions measuring the following eight subscales, rated 0–100 (with higher scores indicating better health status): physical functioning, physical role functioning, emotional role functioning, general health perceptions, social role functioning, bodily pain, vitality, and mental health.

Using the Beck Depression Inventory II (BDI-II) [35, 36], patients assessed 21 items related to symptoms of depression on a 4-point (0–3) scale. Responses were summed to yield a total score that ranged from 0 to 63 (with higher scores indicating more severe depressive symptoms). The widespread pain index (WPI) and symptom severity (SS) scale of the ACR 2010 criteria [8, 37] were assessed, with maximum scores of 19 and 12, respectively.

#### Safety outcomes

Safety was assessed on the basis of the presence or absence and incidence of AEs and adverse drug reactions (ADRs) reported during the treatment phase until the end of the follow-up observation phase. Additionally, laboratory tests (hematology, clinical chemistry, and urinalysis), ECG, body weight, and vital signs were measured. The presence or absence of suicidal tendencies was assessed using the C-SSRS.

### Statistical analyses

Based on clinical data from three previous studies [20–22], the between-group difference in the change in the BPI average pain score from baseline was estimated to be −0.70 between the duloxetine and placebo groups, with a standard deviation of 2.38 for the change in pain score. Therefore, this study required a total of 370 patients to have a power of at least 80 % at a significance level of 0.05 (two-sided). All efficacy analyses were conducted using the full analysis set (FAS), which comprised all randomized patients who received at least one dose of the allocated study drug and had a baseline and at least one postbaseline BPI average pain score. Safety analyses were conducted using the safety analysis set, which was defined as all randomized patients who were administered the study drugs at least once. Unless otherwise noted, the treatment effects were tested at a two-sided significance level of 0.05.

The primary efficacy measure was analyzed using a mixed-effects model repeated-measures (MMRM) approach to compare the change from baseline in BPI average pain score at week 14 of study treatment between the duloxetine and placebo groups. The model included treatment, week, and a treatment × week interaction as fixed effects, as well as the baseline pain score and presence or absence of concomitant major depressive disorder diagnosed as covariates. The change in BPI average pain score from baseline to week 14 of treatment was also compared between the groups by analysis of covariance (ANCOVA) with the baseline value and presence or absence of major depressive disorder as covariates. The missing data at week 14 of treatment were imputed based on the last observation carried forward (LOCF) approach. Post hoc ANCOVA was used for additional sensitivity analyses of the primary efficacy measure. In these post hoc analyses, the baseline observation carried forward (BOCF) or worst observation carried forward (WOCF) values were carried forward to impute missing data instead of the LOCF method being used. The proportions of responders were calculated for patients with a reduction in the BPI average pain score of ≥30 % from baseline to endpoint or ≥50 % from baseline to endpoint, and also for patients with a sustained response. Sustained response was defined as ≥30 % reduction from baseline to endpoint in the BPI average pain score with a 30 % reduction from baseline at an earlier visit (at least 2 weeks prior) and ≥20 % reduction from baseline for every visit in between if there were any intervening visits. These proportions were compared between the groups using a Mantel–Haenszel test adjusted for the allocation factors.

Unless otherwise noted, the MMRM approach was used to evaluate the differences in the changes in other secondary endpoints from baseline between the duloxetine and placebo groups. PGI-I and CGI-I scores were analyzed using a MMRM model without baseline as a covariate. In post hoc analyses, PGI-I and CGI-I at week 14 were compared between the treatment groups using Wilcoxon’s rank-sum test with the LOCF approach. For SF-36, changes from baseline to week 14 of treatment were compared between the duloxetine and placebo groups using the LOCF ANCOVA approach.

Path analysis, which consists of the following two regression models, was performed to estimate a direct analgesic effect relative to an indirect effect on pain reduction through an improvement in depressive symptoms:

$$ {Y}_1={\alpha}_0+{\alpha}_1{X}_1+{\alpha}_2{Y}_2+{\alpha}_3{Z}_1+{\alpha}_4{Z}_2 $$

and

$$ {Y}_2={\beta}_0+{\beta}_1{X}_1+{\beta}_2{Z}_1+{\beta}_3{Z}_2, $$

where *Y*_1_ is the change from baseline in BPI average pain score, *Y*_2_ is the change from baseline in BDI-II total score, *X*_1_ is treatment, *Z*_1_ is baseline BDI-II total score, and *Z*_2_ is baseline BPI average pain score. The direct and indirect effects of the duloxetine were estimated by α_1_ and α_2_ × β_1_, respectively. Then, the contribution (as a percentage) of each effect to the total effect, which was defined as the sum of the direct and indirect effects, was calculated if feasible.

Incidences of AEs and ADRs were compared between the treatment groups using Fisher’s exact test. The AEs and ADRs reported were coded with MedDRA (version 16.1; Medical Dictionary for Regulatory Activities, McLean, VA, USA), and the incidences were tabulated for each preferred term by treatment group.

## Results

### Patient disposition and characteristics

**Fig. 2:**
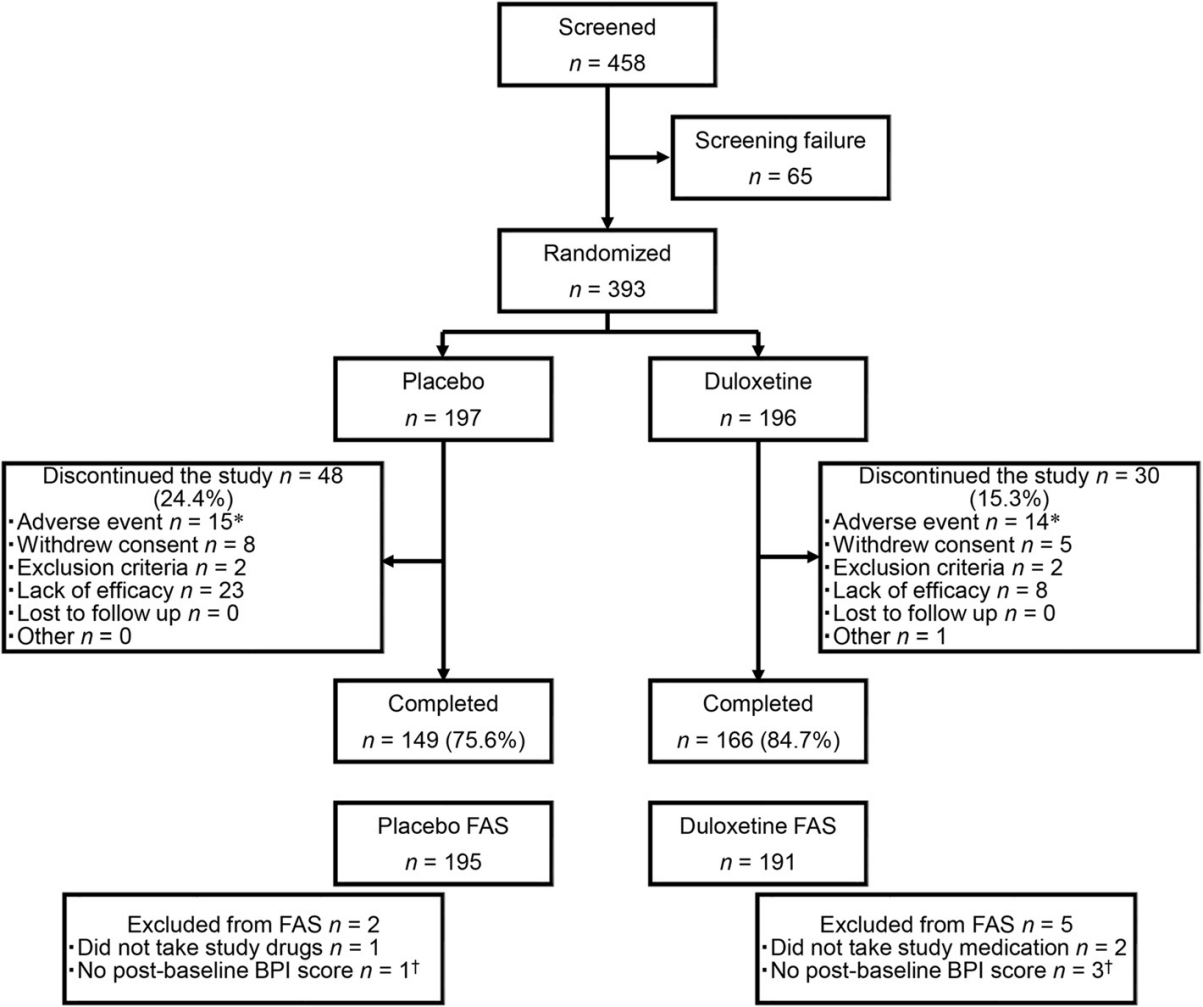
Patient disposition. *Includes discontinuations due to serious adverse events, adverse events, or adverse drug reactions (some patients discontinued because of multiple events). ^†^These patients were included in the safety analysis. *BPI* Brief Pain Inventory; *FAS* full analysis set

In total, 393 patients were enrolled and assigned randomly to receive placebo (*n* = 197) or duloxetine (*n* = 196). In the placebo and duloxetine groups, 2 and 5 patients, respectively, were excluded from the FAS and 149 and 166 patients, respectively, completed the study treatment. Although more patients withdrew because of a lack of efficacy in the placebo group (placebo, 23 [11.7 %]; duloxetine, 8 [4.1 %]), a similar proportion of patients withdrew from each group because of AEs (placebo, 15 [7.6 %]; duloxetine, 14 [7.1 %]) (Fig. 2). The majority of patients were women (83.2 %), with a mean ± SD age of 48.7 ± 11.9 years. Both groups were balanced in terms of baseline demographic characteristics (Table 1).

**Table 1:** Patient characteristics (full analysis set)

	Placebo (n = 195)	Duloxetine (n = 191)	*p* Value
Age, yr	49.5 ± 11.7	47.8 ± 12.0	0.1373^a^
Females	164 (84.1)	157 (82.2)	0.6837
Weight, kg	56.28 ± 10.47	58.00 ± 11.23	0.1219^a^
Height, cm	159.61 ± 7.76	159.41 ± 7.40	0.7922
Major depressive disorder diagnosis	7 (3.6)	8 (4.2)	0.7980
Duration of fibromyalgia, yr	5.7 ± 6.6	5.5 ± 5.9	0.6968
Number of tender points	15.5 ± 2.3	15.4 ± 2.3	0.8740
BPI average pain score (0–10)	6.13 ± 1.35	6.05 ± 1.29	0.5456
FIQ total score (0–100)	56.82 ± 16.14	55.95 ± 16.25	0.5950
BDI-II total score (0–63)	14.89 ± 9.62	15.34 ± 9.73	0.6533

*BDI-II* Beck Depression Inventory II, *BPI* Brief Pain Inventory, *FIQ* Fibromyalgia Impact Questionnaire

Values are means ± standard deviation or n (%). Continuous variables were analyzed using Welch’s *t* test, and categorical variables were analyzed using Fisher’s exact test

^a^Statistically significant at a two-sided significance level of 0.15

### Efficacy and health outcomes

**Fig. 3:**
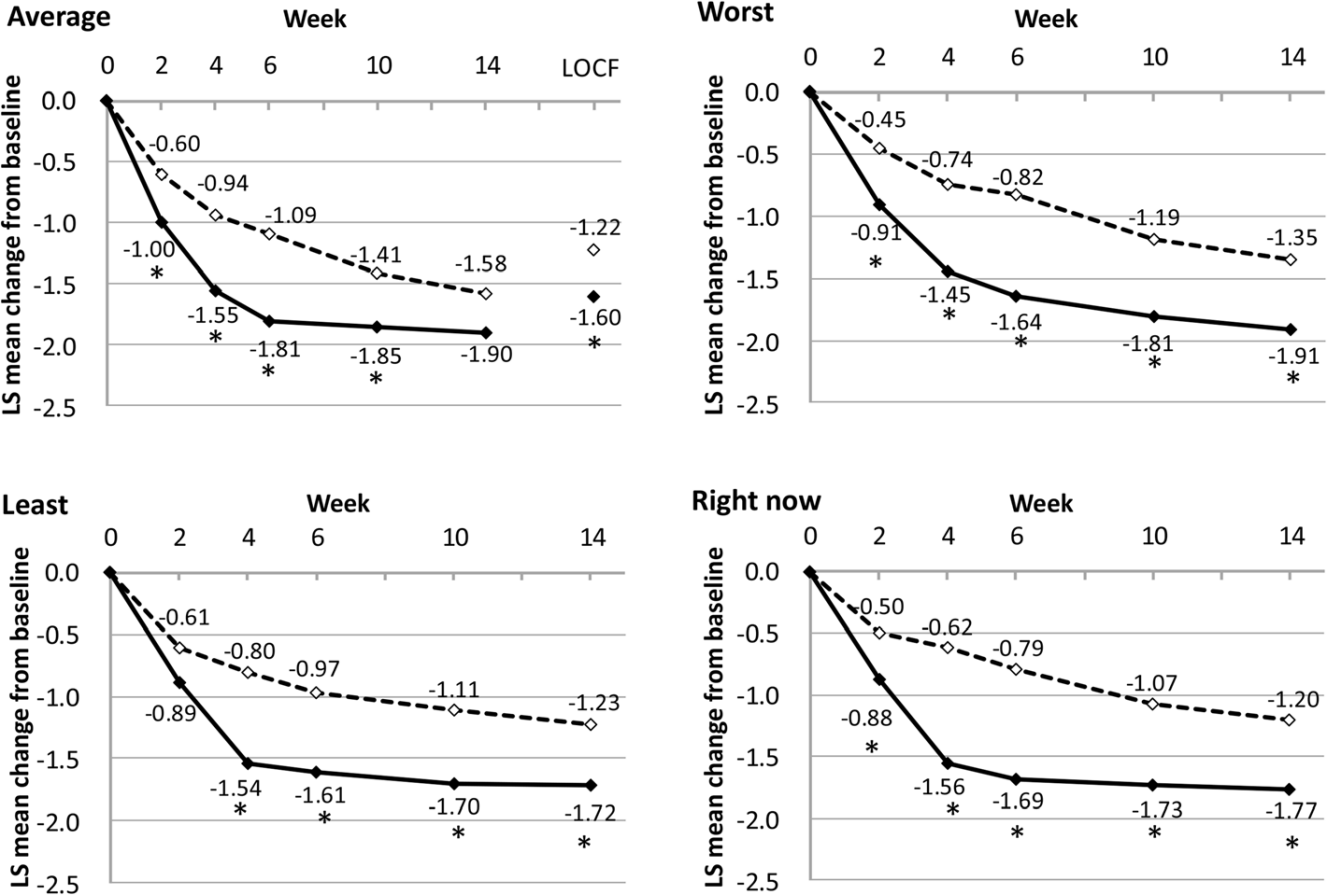
Changes in Brief Pain Inventory average, worst, least, and right now pain scores from baseline. Values are means at each time point. *p < 0.05 vs. placebo. *Dashed lines,* placebo; *black solid lines,* duloxetine. *LOCF* last observation carried forward; *LS* least squares

**Fig. 4:**
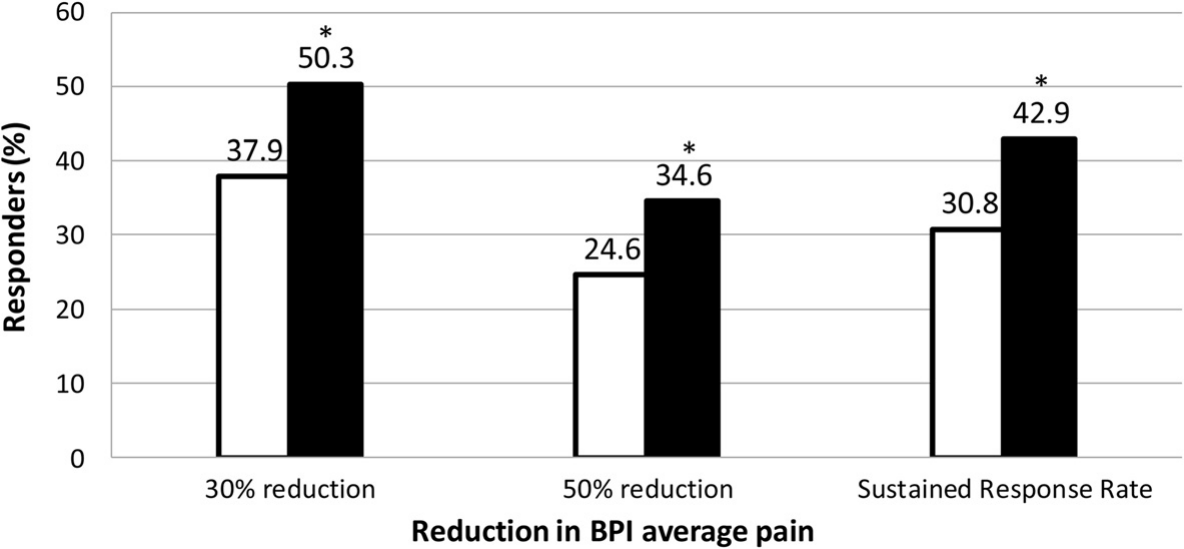
Response rates for the Brief Pain Inventory (BPI) average pain scores at the end of treatment. *p < 0.05 vs. placebo. *Open bars,* placebo; *filled bars,* duloxetine

Although there was no statistically significant difference between the duloxetine and placebo groups in the reduction of the BPI average pain score at week 14 (*p* = 0.0988) in the MMRM analysis, a statistically significant improvement in BPI average pain scores at week 14 was observed for patients treated with duloxetine compared with placebo when we used the LOCF ANCOVA approach (Table 2, Fig. 3). The post hoc BOCF and WOCF analyses also showed that the change in average pain score was significantly greater in the duloxetine group (both *p* = 0.0132) than in the placebo group (Table 2). Analyses of the proportion of responders with pain reduction ≥30 % (*p* = 0.0130) or ≥50 % (*p* = 0.0318) and the sustained response rate (*p* = 0.0139) in the BPI average pain score indicated that the reduction in pain was significantly greater in the duloxetine group than in the placebo group (Fig. 4).

**Table 2:** Changes in efficacy measures from baseline to endpoint or at endpoint

Variable	Placebo (n = 195)		Duloxetine (n = 191)		Difference (95 % CI)	*p* Value
	Baseline	Change	Baseline	Change		
BPI average pain score						
MMRM	6.13 ± 1.35	−1.58 ± 0.23	6.05 ± 1.29	−1.90 ± 0.23	−0.32 (−0.70, 0.06)	0.0988
LOCF	6.13 ± 1.35	−1.22 ± 0.26	6.05 ± 1.29	−1.60 ± 0.26	−0.38 (−0.74, −0.02)	0.0408^a^
BOCF	6.13 ± 1.35	−0.92 ± 0.25	6.05 ± 1.29	−1.38 ± 0.25	−0.45 (−0.81, −0.10)	0.0132^a^
WOCF	6.13 ± 1.35	−0.88 ± 0.26	6.05 ± 1.29	−1.35 ± 0.26	−0.47 (−0.84, −0.10)	0.0132^a^
BPI other pain						
Worst	7.44 ± 1.40	−1.35 ± 0.26	7.36 ± 1.28	−1.91 ± 0.26	−0.56 (−0.99, −0.12)	0.0126^a^
Least	4.46 ± 1.81	−1.23 ± 0.22	4.68 ± 1.70	−1.72 ± 0.22	−0.49 (−0.87, −0.12)	0.0092^a^
Right now	5.90 ± 1.69	−1.20 ± 0.26	5.99 ± 1.52	−1.77 ± 0.26	−0.57 (−1.00, −0.15)	0.0083^a^
Patient diary						
Average pain	5.98 ± 1.39	−1.48 ± 0.18	5.79 ± 1.35	−1.82 ± 0.18	−0.33 (−0.70, 0.03)	0.0755
Worst pain	7.23 ± 1.28	−1.34 ± 0.19	7.05 ± 1.24	−1.81 ± 0.19	−0.47 (−0.88, −0.06)	0.0232^a^
BPI interference scores						
General activities	5.82 ± 2.31	−1.76 ± 0.32	5.82 ± 2.14	−2.22 ± 0.31	−0.46 (−0.98, 0.06)	0.0807
Mood	5.33 ± 2.53	−1.42 ± 0.33	5.65 ± 2.40	−2.17 ± 0.32	−0.75 (−1.29, −0.22)	0.0057^a^
Walking ability	4.08 ± 2.85	−1.29 ± 0.30	4.29 ± 2.73	−1.67 ± 0.29	−0.38 (−0.84, 0.09)	0.1114
Normal work	5.50 ± 2.46	−1.76 ± 0.32	5.61 ± 2.42	−2.18 ± 0.31	−0.42 (−0.94, 0.09)	0.1081
Relationships with people	3.54 ± 2.97	−0.53 ± 0.30	3.90 ± 2.89	−1.09 ± 0.30	−0.55 (−1.04, −0.07)	0.0264^a^
Sleep	5.22 ± 2.91	−1.57 ± 0.36	5.30 ± 2.81	−1.82 ± 0.35	−0.24 (−0.81, 0.32)	0.3959
Enjoyment of life	5.13 ± 2.75	−1.24 ± 0.32	5.12 ± 2.65	−1.90 ± 0.31	−0.66 (−1.18, −0.15)	0.0119^a^
Average of all 7 items	4.95 ± 2.09	−1.44 ± 0.27	5.10 ± 2.07	−1.95 ± 0.27	−0.52 (−0.96, −0.07)	0.0222^a^
FIQ						
Physical functioning	3.85 ± 2.32	−0.37 ± 0.26	3.36 ± 2.35	−0.84 ± 0.25	−0.47 (−0.86, −0.09)	0.0160^a^
Feeling good	7.11 ± 2.73	−0.79 ± 0.41	7.17 ± 2.72	−1.59 ± 0.40	−0.80 (−1.39, −0.21)	0.0082^a^
Missing work	2.44 ± 2.79	−0.48 ± 0.28	2.33 ± 3.09	−0.97 ± 0.27	−0.49 (−0.93, −0.06)	0.0270^a^
Housework	5.86 ± 2.39	−1.69 ± 0.36	5.86 ± 2.45	−2.14 ± 0.35	−0.45 (−0.97, 0.08)	0.0932
Pain	7.01 ± 1.67	−1.76 ± 0.35	6.83 ± 1.52	−2.37 ± 0.34	−0.62 (−1.11, −0.12)	0.0148^a^
Fatigue	7.27 ± 2.08	−1.45 ± 0.35	7.08 ± 1.97	−1.96 ± 0.34	−0.52 (−1.03, 0.00)	0.0479^a^
Morning tiredness	6.81 ± 2.41	−1.68 ± 0.39	6.86 ± 2.40	−1.80 ± 0.37	−0.13 (−0.69, 0.44)	0.6618
Stiffness	6.26 ± 2.56	−1.59 ± 0.35	6.20 ± 2.57	−2.10 ± 0.34	−0.51 (−1.03, 0.02)	0.0577
Anxiety	5.43 ± 2.67	−1.18 ± 0.36	5.33 ± 2.59	−1.86 ± 0.35	−0.68 (−1.20, −0.15)	0.0114^a^
Depression	4.79 ± 2.71	−0.96 ± 0.35	4.91 ± 2.76	−1.62 ± 0.34	−0.66 (−1.18, −0.14)	0.0129^a^
Total score	56.82 ± 16.14	−13.05 ± 2.65	55.95 ± 16.25	−18.41 ± 2.57	−5.35 (−9.26, −1.45)	0.0073^a^
SF-36						
Physical functioning	62.51 ± 19.82	3.04 ± 2.15	63.72 ± 18.75	7.40 ± 2.13	4.36 (1.35, 7.37)	0.0046^a^
Physical role limitations	49.13 ± 25.60	0.44 ± 2.98	49.25 ± 25.57	8.20 ± 2.96	7.76 (3.57, 11.94)	0.0003^a^
Bodily pain	36.60 ± 11.71	5.28 ± 2.08	36.53 ± 12.40	10.95 ± 2.07	5.67 (2.76, 8.59)	0.0002^a^
General health perceptions	38.76 ± 14.77	3.31 ± 1.94	39.37 ± 17.67	6.55 ± 1.92	3.25 (0.53, 5.96)	0.0192^a^
Vitality	31.96 ± 18.80	3.35 ± 2.53	32.43 ± 21.03	10.05 ± 2.51	6.70 (3.15, 10.25)	0.0002^a^
Social functioning	55.71 ± 26.54	3.28 ± 3.06	55.76 ± 27.53	10.32 ± 3.04	7.04 (2.74, 11.34)	0.0014^a^
Emotional role limitations	61.24 ± 26.80	−3.63 ± 3.36	60.34 ± 29.16	5.50 ± 3.35	9.12 (4.41, 13.83)	0.0002^a^
Mental health	56.10 ± 19.84	−2.00 ± 2.52	55.50 ± 18.85	5.91 ± 2.51	7.91 (4.39, 11.43)	<0.0001^a^
CGI-I^b^	–	3.27 ± 0.16	–	2.83 ± 0.15	−0.44 (−0.71, −0.18)	0.0012^a^
PGI-I^b^	–	3.32 ± 0.16	–	2.83 ± 0.16	−0.49 (−0.76, −0.22)	0.0003^a^
BDI-II score (0–63)	14.89 ± 9.62	−1.19 ± 0.85	15.34 ± 9.73	−4.09 ± 0.84	−2.90 (−4.37, −1.44)	0.0001^a^
ACR 2010						
WPI (0–19)	12.08 ± 3.57	−1.06 ± 0.60	12.14 ± 3.58	−2.34 ± 0.58	−1.28 (−2.12, −0.44)	0.0029^a^
SS (0–12)	6.59 ± 1.88	−1.00 ± 0.28	6.60 ± 1.82	−1.37 ± 0.27	−0.36 (−0.79, 0.06)	0.0906
Total (0–31)	18.67 ± 4.53	−2.24 ± 0.77	18.74 ± 4.36	−3.88 ± 0.74	−1.64 (−2.74, −0.54)	0.0037^a^

*ACR* American College of Rheumatology, *BDI-II* Beck Depression Inventory II, *BOCF* baseline observation carried forward, *BPI* Brief Pain Inventory, *CGI-I* Clinical Global Impressions–Global Improvement, *CI* confidence interval, *FIQ* Fibromyalgia Impact Questionnaire, *LOCF* last observation carried forward; *MMRM* mixed-effects model repeated measures, *PGI-I* Patient Global Impression of Improvement, *SF-36* 36-Item Short Form Survey; *SS* symptom severity; *WOCF* worst observation carried forward, *WPI* widespread pain index

Values are means ± SD (baseline) or least-squares means ± SE (change)

^a^Statistically significant at a two-sided level of 0.05

^b^Mean scores at the end of treatment based on a 7-point scale

**Fig. 5:**
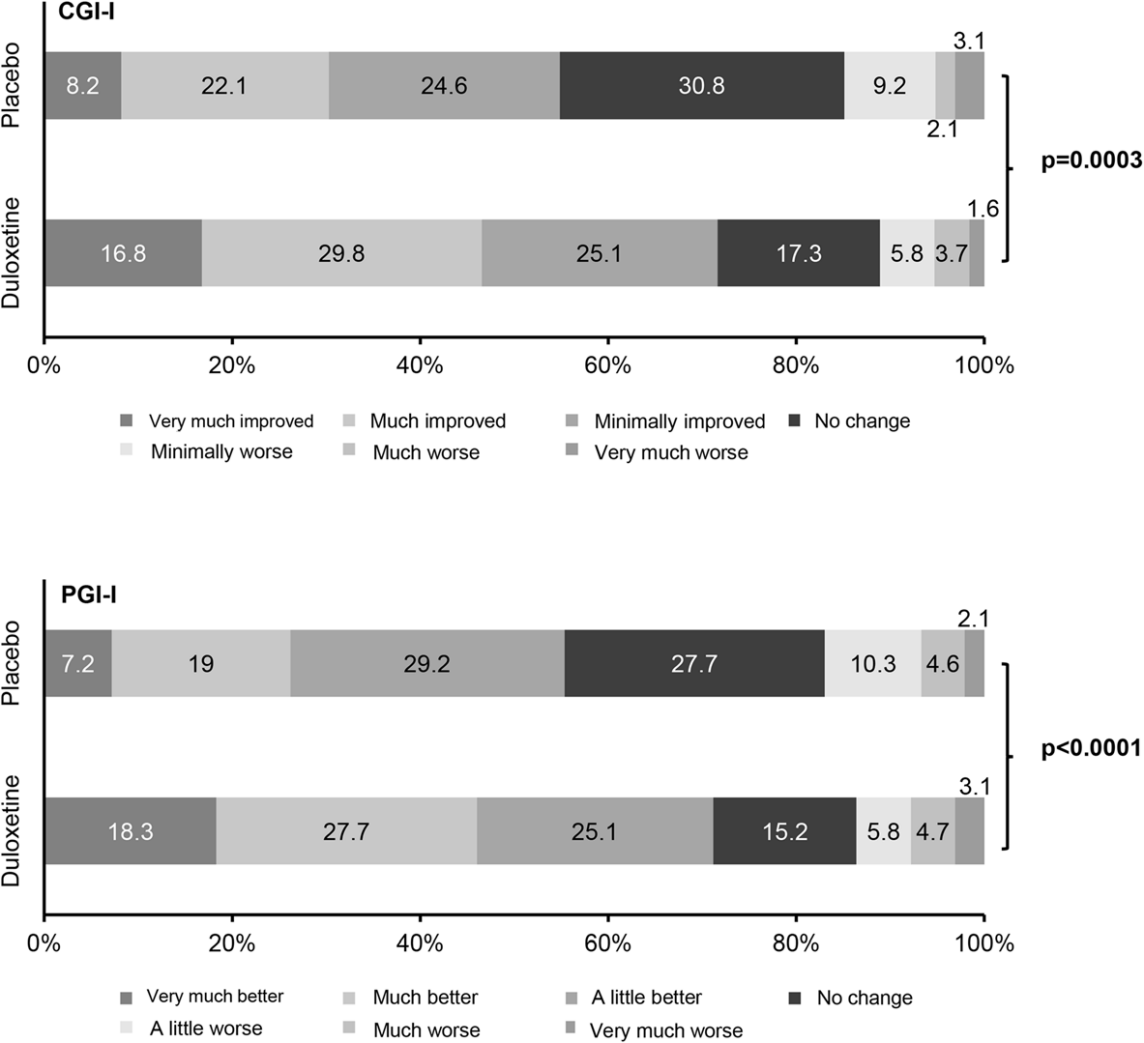
Patient Global Impression of Improvement (PGI-I) and Clinical Global Impressions–Global Improvement (CGI-I) ratings at the end of treatment. PGI-I and CGI-I scores were recorded on a 7-point scale where 1 = very much better/improved, 2 = much better/improved, 3 = a little better/improved, 4 = no change, 5 = a little worse, 6 = much worse, and 7 = very much worse. *p < 0.05 vs. placebo

Regarding the secondary outcome measures of pain, duloxetine treatment was associated with significant reductions in BPI worst pain, BPI least pain, BPI pain right now, worst pain in patient diary, FIQ pain score, and SF-36 bodily pain score (Table 2). General illness, measured using the CGI-I and PGI-I, was also significantly improved in the duloxetine group in terms of both the mean scores (Table 2) and the proportions of clinicians and patients reporting improvements in CGI-I and PGI-I (Fig. 5). Duloxetine treatment was also associated with a significant improvement in the patients’ quality of life (QoL) as measured by the patient-related health outcomes BPI interference, FIQ score, and SF-36 score (Table 2). A significant improvement with a between-group difference of −1.64 (95 % CI, −2.74, −0.54; *p* = 0.0037) was also observed in the total WPI and SS scores (Table 2). The proportion of patients matching ACR 2010 criteria (WPI ≥7 and SS ≥5 or WPI 3–6 and SS ≥9) in the FAS was 85.0 %.

The path analysis of BPI average pain score performed to estimate the direct analgesic effect indicated that treatment effect at the study endpoint did not correspond to a sizable portion of the total effect (Table 3). The direct reduction of the BPI average pain score by duloxetine accounted for 28.3 % of the total treatment effect, whereas the indirect treatment effect through improvement of depressive symptoms accounted for 71.7 % of the total treatment effect.

**Table 3:** Path analysis for direct analgesic effect on the reduction of the BPI average pain score

Week	Direct effect	Indirect effect
Week 2^a^	84.3	15.7
Week 4^a^	83.8	16.2
Week 6^a^	63.1	36.9
Week 10^a^	43.2	56.8
Week 14^a^	41.6	58.4
Endpoint^b^	28.3	71.7

Values are the percentages of direct and indirect effects

^a^Observed case

^b^Last observation carried forward

### Safety and tolerability

There were no deaths reported during the study. Two serious AEs (pneumonia and asthma) were reported in one patient in the placebo group. One serious AE (liver injury) was reported in one patient in the duloxetine group, and the investigator could not exclude the possibility of a relationship between the AE and the study drug. That patient recovered after discontinuation of the study drug. The number of patients who discontinued treatment because of AEs was similar in both groups, with 15 and 14 patients in the placebo and duloxetine groups, respectively. Somnolence (placebo, 10.7 % vs. duloxetine, 26.3 %), nausea (4.6 % vs. 21.6 %), constipation (4.1 % vs. 14.9 %), decreased appetite (0.5 % vs. 6.7 %), and dizziness (1.0 % vs. 5.7 %) were significantly more frequent in the duloxetine group than in the placebo group (Table 4). Most of the adverse effects were mild in severity, and patients either recovered immediately or promptly after treatment discontinuation. No distinct changes attributable to duloxetine were observed in laboratory test results, blood pressure and pulse rate, body weight, or ECG. None of the patients had an apparent suicide risk according to the C-SSRS.

**Table 4:** Adverse events (safety analysis set)

	Placebo (n = 196)	Duloxetine (n = 194)	*p* Value^a^
AEs	123 (62.8)	148 (76.3)	0.0042^b^
ADRs	70 (35.7)	125 (64.4)	<0.0001^b^
Serious AEs	1 (0.5)	1 (0.5)	1.000
Serious ADRs	0 (0.0)	1 (0.5)	0.4974
Discontinuations due to AEs	15 (7.7)	14 (7.2)	1.000
Discontinuations due to ADRs	10 (5.1)	14 (7.2)	0.4077
AEs by preferred term			
Somnolence	21 (10.7)	51 (26.3)	<0.0001^b^
Nausea	9 (4.6)	42 (21.6)	<0.0001^b^
Constipation	8 (4.1)	29 (14.9)	0.0002^b^
Nasopharyngitis	29 (14.8)	26 (13.4)	0.7715
Dry mouth	7 (3.6)	14 (7.2)	0.1218
Decreased appetite	1 (0.5)	13 (6.7)	0.0008^b^
Dizziness	2 (1.0)	11 (5.7)	0.0112^b^
Headache	6 (3.1)	9 (4.6)	0.4437
Fatigue	6 (3.1)	9 (4.6)	0.4437
Diarrhea	7 (3.6)	8 (4.1)	0.7990

*ADR* adverse drug reaction, *AE* adverse event

Values are n (%)

^a^Fisher’s exact test

^b^Statistically significant at a two-sided level of 0.05

## Discussion

### Analgesic effect of duloxetine on fibromyalgia

In this randomized, multicenter, double-blind, placebo-controlled phase III trial of duloxetine in Japanese patients with fibromyalgia, the MMRM analysis of the primary efficacy measure, in which we compared the change in BPI average pain scores from baseline to week 14 between duloxetine and placebo groups, did not demonstrate superiority of duloxetine over placebo. However, when the change in BPI average pain score at each evaluation time point was examined, a significant improvement was observed at all the evaluation time points (from weeks 2 to 10) in the duloxetine group compared with the placebo group.

The efficacy and safety of duloxetine in the treatment of fibromyalgia was previously investigated in four randomized, double-blind, placebo-controlled trials in other countries [17, 20–22]. A pooled analysis of the results of these trials showed that 12 weeks of treatment with duloxetine significantly reduced pain compared with placebo beginning in the first week of treatment and that this reduction continued at each subsequent week throughout the 12 weeks of therapy [18].

Furthermore, analysis of the change in BPI average pain score from baseline to week 14 using ANCOVA (with missing data imputed by LOCF) showed a significant improvement in the duloxetine group compared with the placebo group. These results are consistent with those of earlier international studies [20, 21] in which researchers evaluated once-daily duloxetine 60 mg and reported a significant improvement in the change from baseline to study endpoint (using LOCF) in the duloxetine group compared with the placebo group, based on ANCOVA. In these countries, including the United States, once-daily duloxetine 60 mg was approved for the treatment of fibromyalgia on the basis of such clinical study data.

Moreover, in post hoc analyses of the results of our present study, the change from baseline to week 14 in BPI average pain scores was compared when missing data due to discontinuations were imputed using the BOCF or WOCF methods. These results indicate that, with either of the missing data imputation methods, a significant improvement was observed in the duloxetine group compared with the placebo group. In this clinical trial, there were more patients who discontinued the trial because of an inadequate improvement in the BPI average pain score or exacerbation of pain in the placebo group (11.7 %) compared with the duloxetine group (4.1 %). Therefore, the MMRM analysis might introduce a bias toward greater improvement in BPI average pain score in the placebo group than in the duloxetine group.

Of the seven pain-related items among the secondary efficacy measures, six (BPI pain scores [worst, least, and pain right now], worst pain reported in the patient diary, FIQ pain score, and SF-36 [bodily pain]) showed a significant improvement from baseline to week 14 of treatment in the duloxetine group compared with the placebo group. Although the superiority of duloxetine over placebo was not demonstrated in the MMRM analysis of the primary efficacy measure, a significant improvement in the duloxetine group compared with the placebo group was observed in the secondary and post hoc analyses and several other secondary efficacy measures. These results suggest that treatment with duloxetine could be associated with improvement in pain relief in Japanese patients with fibromyalgia.

Moreover, the 30 %, 50 %, and sustained pain reductions based on the change at endpoint in the BPI average pain score were significantly higher in the duloxetine group than in the placebo group. According to the Initiative on Methods, Measurement, and Pain Assessment in Clinical Trials (IMMPACT) recommendations, patients with a clinically relevant reduction in the assessment scale of 30–50 % compared with baseline are considered responders [38]. Therefore, the rate of responders was significantly higher in the duloxetine group than in the placebo group in the present trial. Considering that we assessed patients with moderate or severe fibromyalgia, with characteristics similar to those of the patients enrolled in Japanese epidemiological studies [10], duloxetine treatment might benefit such individuals.

The path analysis performed in this study suggested that the direct effect of duloxetine on the reduction of the BPI average pain was smaller than the indirect treatment effect, as evidenced by the improvement in depressive symptoms at study endpoint. The changes in both BPI average pain score and BDI-II score indicated improvements in these symptoms. Because the between-groups difference at the last evaluation was large for BDI-II and small for BPI average pain score, we estimate that the contribution of improved mood (i.e., the indirect analgesic effect) to the whole analgesic effect was high. In the present clinical study, however, the proportion of patients with a complication of major depressive disorder was small compared with the proportions observed in other studies: major depressive disorder was diagnosed in only eight patients (4.2 %) in the duloxetine group and seven patients (3.6 %) in the placebo group in our study. Thus, an evaluation of exactly how much of the analgesic effect of duloxetine was due to its antidepressant effect was considered difficult. A subgroup analysis using LOCF ANCOVA was performed to examine the potential effects of the degree of depressive symptoms (divided into a minimal depression group with baseline BDI-II scores of 0–13 points and a group with scores ≥14 points according to the BDI-II manual [35]) and the presence or absence of complications of major depressive disorder on the improvement of pain. The between-groups difference in the change in the BPI average pain score tended to be greater in patients with a baseline BDI-II score <14 (between-groups difference, −0.51; 95 % CI, −1.03, 0.02; *p* = 0.0586) than in patients with a baseline BDI-II score of ≥14 (between-groups difference, −0.27; 95 % CI, −0.78, 0.24; *p* = 0.2946). In patients without major depressive disorder, the reduction in the BPI average pain score was significantly greater in the duloxetine group (between-groups difference, −0.38; 95 % CI, −0.75, 0.00; *p* = 0.0494). Therefore, treatment with duloxetine could be associated with an improvement in patients with very mild depressive symptoms as well as in those without major depressive disorder.

### Changes in associated symptoms of fibromyalgia

Fibromyalgia is characterized by widespread body pain as well as a variety of symptoms that affect overall health status and QoL. Therefore, PGI-I and CGI-I scores were assessed for comprehensive evaluation of illness improvement from baseline to 14 weeks of treatment. The SF-36 and FIQ were used, in addition to the BPI pain interference scale, to assess the overall impact of fibromyalgia on health status. Because fibromyalgia is associated with neuropsychiatric symptoms, the BDI-II was used to assess improvement in these symptoms. In this study, illness improvement (PGI-I and CGI-I scores) was significantly higher in the duloxetine group than in the placebo group. The BPI interference scores for mood, relationships with people, and enjoyment of life, as well as the average score for all seven items, were significantly improved in the duloxetine group compared with the placebo group. These improvements in the BPI interference scores are similar to the results of a study conducted in Japanese patients with diabetic peripheral neuropathic pain [24] who presented improvements in the scores for walking ability, relationships with people, sleep, and enjoyment of life items and improvement of −0.48 in the average score of the seven BPI interference items. In our study, compared with the placebo group, the duloxetine group showed significant improvements in seven subscales of the FIQ (motor dysfunction, emotional well-being, number of days absent from work or housework, pain, fatigue, anxiety, and depressive state) and in all eight subscales of the SF-36, indicating that duloxetine treatment could be associated with improvement in QoL in this cohort of patients. We also observed a significant improvement in the BDI-II score, which indicates that duloxetine improved psychiatric symptoms in our patient cohort.

The present study suggests that duloxetine treatment could be associated with a reduction in the pain associated with fibromyalgia and the improvement of related symptoms. Because of the wide array of clinical manifestations of fibromyalgia, the disease can considerably deteriorate the QoL of patients. On the basis of the present findings, duloxetine could potentially improve the QoL of patients as well as ameliorate depressive symptoms by improving the symptoms associated with fibromyalgia.

### American College of Rheumatology 2010 criteria

Compared with the placebo group, the duloxetine group showed a significant improvement in WPI and a trend toward a numerical improvement in SS, indicating an improvement of clinical symptoms. This result is consistent with the significant improvement in associated symptoms of fibromyalgia and in several items of the QoL assessment in patients who received duloxetine in this study. Moreover, 85.0 % of patients in this study matched the ACR 2010 criteria for fibromyalgia, which is consistent with the findings reported by Usui et al. [37]. The above findings suggest the usefulness of ACR 2010 in the diagnosis of fibromyalgia and the severity assessment of clinical symptoms in clinical studies of fibromyalgia. Because only a very limited number of studies evaluating the SS and WPI have been reported to date, more data are needed to confirm the significance of the results obtained in the present study.

### Onset of effect by once-daily dosing

Generally, patient compliance has been found to increase as dosing frequency decreases [39]. Currently, the only drug indicated for the treatment of fibromyalgia in Japan is recommended in a twice-daily administration regimen. However, on the basis of the results of this and previous studies, once-daily dosing of duloxetine appears to be sufficient to improve pain and may improve QoL outcomes, which may contribute to greater patient compliance and convenience.

### Safety

The results of the present short-term study indicate that there were no significant safety concerns related to duloxetine treatment. Somnolence, nausea, constipation, decreased appetite, and dizziness were significantly more frequent in the duloxetine group than in the placebo group, which is consistent with previously reported AEs among patients with approved indications, such as major depressive disorder [23] and diabetic peripheral neuropathic pain [24]. In addition, comprehensive safety analysis of studies done in other countries also revealed that the most common AEs seen during duloxetine treatment were similar to those observed during treatment with duloxetine for other indications. Most AEs were mild to moderate in severity and were transient [19]. Therefore, in the present study, there were no new findings that would change the existing duloxetine safety profile. It will be important to confirm the long-term safety of duloxetine because patients with chronic pain, such as those with fibromyalgia, generally undergo long-term treatment. We have implemented an open-label, long-term extension trial of this study to continue evaluating the safety of duloxetine.

### Limitations

Some limitations of this study should be mentioned: we excluded patients with psychiatric disorders other than depression, and we did not assess the long-term efficacy and safety of duloxetine.

## Conclusions

Although the MMRM analysis did not demonstrate superiority of duloxetine over placebo, duloxetine treatment was associated with improved outcomes in secondary and post hoc analyses of the mean change of the BPI average pain score and most of the secondary outcomes, including analgesia and QoL. No significant safety concerns were reported during the duloxetine treatment. These results suggest that treatment with duloxetine could be associated with improvement in pain relief and QoL in Japanese patients with fibromyalgia.

## Appendix

### List of institutional review boards

- Institutional Review Board of Amagasaki Chuo Hospital
- Institutional Review Board of Hanna Hospital
- Institutional Review Board of Haradoi Hospital
- Institutional Review Board of Himeno Tomomi Clinic
- Institutional Review Board of Hiroshima Clinic
- Institutional Review Board of Juntendo University Nerima Hospital
- Institutional Review Board of Kayaba Dermatology Clinic
- Institutional Review Board of Munakata Yasuhiko Clinic
- Institutional Review Board of Nakayama Rheumatism and Allergy Clinic
- National Hospital Organization Shimoshizu Hospital Institutional Review Board
- Oita Central Institutional Review Board
- Sendai Institutional Review Board
- Institutional Review Board of Shinsapporo Seiryou Hospital
- Institutional Review Board of Shinagawa East One Medical Clinic
- Institutional Review Board of Shinonoi General Hospital
- Institutional Review Board of Sone Clinic
- Japanese Association for the Promotion of State of the Art in Medicine Institutional Review Board
- Institutional Review Board of Tokyo Medical University Hachioji Medical Center
- Institutional Review Board of Tokyo-Eki Center-Building Clinic
- Institutional Review Board of Tomisaka Clinic
- Institutional Review Board of Yokohama Minami Kyousai Hospital
- Institutional Review Board of Yokohama Minoru Clinic
- St. Marianna University Group Institutional Review Board

## Abbreviations

ACR: American College of Rheumatology
ADR: Adverse drug reaction
AE: Adverse event
ANCOVA: Analysis of covariance
BDI-II: Beck Depression Inventory II
BOCF: Baseline observation carried forward
BPI: Brief Pain Inventory
CGI-I: Clinical Global Impressions–Global Improvement
CI: Confidence interval
CNS: Central nervous system
C-SSRS: Columbia-Suicide Severity Rating Scale
ECG: Electrocardiogram
FAS: Full analysis set
FIQ: Fibromyalgia Impact Questionnaire
5-HT: Serotonin
IMMPACT: Initiative on Methods, Measurement, and Pain Assessment in Clinical Trials
LOCF: Last observation carried forward
LS: Least squares
MMRM: Mixed-effects model with repeated measures
NA: Noradrenaline
PGI-I: Patient Global Impression of Improvement
QoL: Quality of life
SF-36: 36-Item Short Form Survey
SS: Symptom severity
WOCF: Worst observation carried forward
WPI: Widespread pain index
